# Supplementary material for: Mortality for Lung Cancer among PVC Baggers Employed in the Vinyl Chloride Industry
Source: Int J Environ Res Public Health. 2022 May 20;19(10):6246. doi: 10.3390/ijerph19106246 (PMC9141742; doi:10.3390/ijerph19106246)
Supplement: Supplementary file 1 [file ijerph-19-06246-s001.zip › ijerph-1700205-supplementary.pdf]

Supplementary Table S1. Distribution of the study variables by available/imputed data on smoking habit status.

	**Overall**, N = 1658 [1]	**Interviewed**, N = 848 [1]	**Non interviewed** N = 810 [1]	***p*-value** [2]
**Age at first exposure**, n (%)				0.068
[17.2,23.3] years	415 (25%)	192 (23%)	223 (28%)	
(23.3,27.4] years	414 (25%)	220 (26%)	194 (24%)	
(27.4,33.3] years	414 (25%)	208 (25%)	206 (25%)	
(33.3,57.9] years	415 (25%)	228 (27%)	187 (23%)	
**Year at first exposure**, n (%)				0.014
[1951,1961]	428 (26%)	237 (28%)	191 (24%)	
(1961,1968]	407 (25%)	223 (26%)	184 (23%)	
(1968,1972]	412 (25%)	197 (23%)	215 (27%)	
(1972,1986]	411 (25%)	191 (23%)	220 (27%)	
**Duration of work**, n (%)				<0.001
[0.04,6.21] years	415 (25%)	154 (18%)	261 (32%)	
(6.21,12.4] years	418 (25%)	203 (24%)	215 (27%)	
(12.4,18.6] years	413 (25%)	236 (28%)	177 (22%)	
(18.6,31.7] years	412 (25%)	255 (30%)	157 (19%)	
**Time since dismissal to 31 July 1999 or death**, n (%)				<0.001
(0.00,14.1] years	814 (49%)	494 (58%)	320 (40%)	
(14.1,15.1] years	37 (2.2%)	20 (2.4%)	17 (2.1%)	
(15.1,23.1] years	424 (26%)	165 (19%)	259 (32%)	
(23.1,43.1] years	383 (23%)	169 (20%)	214 (26%)	
**Life status at 31 July 1999**, n (%)				<0.001
Alive	1,404 (85%)	776 (92%)	628 (78%)	
Deceased for lung cancer	44 (2.7%)	38 (4.5%)	6 (0.7%)	
Deceased for cardiovascular disease	45 (2.7%)	5 (0.6%)	40 (4.9%)	
Deceased for liver cancer	16 (1.0%)	9 (1.1%)	7 (0.9%)	
Deceased for other causes	149 (9.0%)	20 (2.4%)	129 (16%)	
**Time since 31 July 1999 to last follow-up**, n (%)				<0.001
Deceased before the previous follow-up	254 (15%)	72 (8.5%)	182 (22%)	
(0,6.27] years	161 (9.7%)	45 (5.3%)	116 (14%)	

(6.27,17.7] years	1,243 (75%)	731 (86%)	512 (63%)	
**Life Status at the last follow-up**, n (%)				0.8
Alive	1,181 (71%)	595 (70%)	586 (72%)	
Deceased for lung cancer	56 (3.4%)	28 (3.3%)	28 (3.5%)	
Deceased for cardiovascular disease	127 (7.7%)	71 (8.4%)	56 (6.9%)	
Deceased for liver cancer	41 (2.5%)	23 (2.7%)	18 (2.2%)	
Deceased for other causes	253 (15%)	131 (15%)	122 (15%)	
**Task as a bagger**, n (%)				0.7
Never	1,450 (87%)	745 (88%)	705 (87%)	
Ever	120 (7.2%)	62 (7.3%)	58 (7.2%)	
Exclusive	88 (5.3%)	41 (4.8%)	47 (5.8%)	

[1] n (%), [2] Pearson's Chi-squared test.

Supplementary Table S2. Adjusted* multivariate Mortality Rate Ratios (MRR) for lung cancer estimated by Poisson regression and 95% Confidence Intervals (95% CI) for the complete cases and for the imputed datasets. The value of γ represents the fraction of information missing due to nonresponse.

**Complete cases (n=848)**		**MRR**	**95% CI**	
Duration of the task as Bagger	0	1.00		
	<3 years	1.49	(0.57-3.23)	
	≥3 years	2.13	(0.92-4.28)	
Smoking habit	No smoker (reference)	1.00		
	<15 cigarettes	4.46	(1.43-19.5)	
	≥15 cigarettes	22.9	(8.39-94.1)	
**Entire dataset (n=1658)**		**MRR**	**95% CI**	γ
Duration of the task as Bagger	0	1.00		
	<3 years	1.22	(0.61-2.47)	2.22%
	≥3 years	1.72	(0.88-3.37)	2.90%
Smoking habit	No smoker (reference)	1.00		
	<15 cigarettes	1.66	(0.69-4.02)	42.1%
	≥15 cigarettes	5.63	(2.67-11.9)	38.0%

* Adjusted for 10 years age-class (≤50, 51-60, 61-70,71-80, >80) and calendar period (≤1980, 1981-1990, 1991-2000, 2001-2010, >2010), and latency (≤10, 11-20, 21-30, >30).
